# Fasudil, a Rho-Kinase Inhibitor, Exerts Cardioprotective Function in Animal Models of Myocardial Ischemia/Reperfusion Injury: A Meta-Analysis and Review of Preclinical Evidence and Possible Mechanisms

**DOI:** 10.3389/fphar.2018.01083

**Published:** 2018-10-01

**Authors:** Yue-yue Huang, Jian-ming Wu, Tong Su, Song-yue Zhang, Xiao-ji Lin

**Affiliations:** ^1^Department of Internal Medicine, The Second Affiliated Hospital and Yuying Children's Hospital of Wenzhou Medical University, Wenzhou, China; ^2^Department of Dermatovenereology, The Second Affiliated Hospital and Yuying Children's Hospital of Wenzhou Medical University, Wenzhou, China; ^3^Department of Pediatric Cardiology, The Second Affiliated Hospital and Yuying Children's Hospital of Wenzhou Medical University, Wenzhou, China

**Keywords:** fasudil, myocardial ischemia/reperfusion injury, efficacy, mechanisms, meta-analysis, systematic review

## Abstract

Fasudil, a Rho-kinase inhibitor, has shown outstanding therapeutic effects against cerebral vasospasm after subarachnoid hemorrhage (SAH) in humans. Studies show various biological effects of fasudil in the cardiovascular system. We conducted a preclinical systematic review to determine the efficacy and possible mechanisms of fasudil on animal models of myocardial ischemia/reperfusion (I/R) injury. Nineteen studies involving 400 animals were identified after searching 8 databases for articles published till June 2018. The methodological quality was assessed by the Collaborative Approach to Meta-Analysis and Review of Animal Data from Experimental Studies (CAMARADES) 10-item checklist. The data were analyzed using Rev-Man 5.3 software, and the score of study quality ranged from 3 to 6 points. Compared to the control group, fasudil treated animals showed reduced myocardial infarct size (*P* < 0.05), lower levels of cardiac enzymes (*P* < 0.05) and cardiac troponin T (*P* < 0.05), improved systolic and diastolic functions (*P* < 0.05), and increased degree of decline in the ST-segment (*P* < 0.05). The possible mechanisms of fasudil action against myocardial I/R injury are improvement in coronary vasodilation, inhibition of apoptosis and oxidative stress, relieving inflammation, and reduction in endoplasmic reticulum stress and metabolism. In conclusion, fasudil exerts a cardio-protective function through multiple signaling pathways in animal models of myocardial I/R injury.

## Introduction

Acute myocardial infarction (AMI) caused by acute cardiomyocyte ischemia is one of the main etiologies of morbidity and mortality worldwide (Benjamin et al., 2018). Experimental and proof-of-concept clinical trials have shown that the infarct is a result of injuries induced by ischemia and reperfusion (Zhao et al., 2003; Staat et al., 2005; Ibáñez et al., 2015). Therefore, restoring blood flow in a timely manner and preventing myocardial ischemia/reperfusion (I/R) injury are crucial to rescuing an ischemic myocardium (Ribas et al., 2017). Reperfusion strategies such as thrombolysis, percutaneous coronary intervention (PCI) and coronary artery bypass grafting (CABG) have been developed in recent years, and significantly reduce mortality and infarct size and improve left ventricular function (Heusch and Gersh, 2017). However, abrupt restoration of coronary flow can lead to adverse events such as reversible impairment of myocardial contractility (myocardial stunning), ventricular arrhythmias, and microvascular dysfunction (Heusch and Gersh, 2017), thereby reducing the benefits of reperfusion therapies (Yellon and Hausenloy, 2007). Several therapeutic strategies against myocardial I/R injury have been developed over the years and have shown encouraging results in pre-clinical studies (Chun et al., 2011). However, they have not been successfully translated into notable clinical benefits (Cung et al., 2015; Hausenloy et al., 2015; Meybohm et al., 2015; Bochaton and Ovize, 2018). Therefore, it is vital to develop novel cardio-protective strategies to improve myocardial function in order to reduce the risk of myocardial I/R injury.

Rho-kinase activity is associated with several cardiovascular diseases including myocardial I/R injury (Nunes et al., 2010; Satoh et al., 2011). A previous study (Kitano et al., 2014) showed that Rho-kinase is activated during myocardial reperfusion, indicating that the inhibition of Rho-kinase activity is a potential target in suppressing the reperfusion injury salvage kinase (RISK) pathway and prevent myocardial I/R injury. Fasudil, a Rho-kinase inhibitor, has shown remarkable therapeutic effect against cerebral vasospasm after subarachnoid hemorrhage (SAH) in humans (Masaoka et al., 2001). Recent studies show that fasudil exerts its cardio-protective effects by relieving inflammation/oxidative stress (Shimokawa and Takeshita, 2005; Ma et al., 2011), inhibiting apoptosis (Tong and Zhang, 2007) and fibrosis (Xu et al., 2017), and improving coronary blood flow (Aizawa et al., 2012). However, its efficacy and mechanism of action against experimental myocardial I/R injury have not been systematically evaluated yet, which limits its clinical use. A systematic review of animal studies has proven invaluable to clarify the mechanisms, etiology and therapy of human diseases (Sena et al., 2014). Therefore, we reviewed the pre-clinical studies on fasudil, and evaluated its efficacy and possible mechanism of action in myocardial I/R injury.

## Methods

### Search strategies

Preferred Reporting Items for Systematic Review and Meta-Analyses (PRISMA) statement were abided (Stewart et al., 2015). Experimental studies estimating the efficacy of fasudil in animal models of MI were systematically searched from EMBASE, PubMed, Cochrane Library, Web of Science, Wangfang database, China National Knowledge Infrastructure (CNKI), vip database (VIP), China Biology Medicine disc (CBM) from inception to the end of June 2018. The key words were used as follows: “fasudil (MeSH Terms) OR fasudil (Title/Abstract)” AND “myocardial infarction OR myocardial ischemia OR myocardial I/R OR myocardial I/R injury.” Moreover, reference lists of potential studies were searched for relevant studies.

### Eligibility criteria and data extraction

To prevent bias, the following inclusion criteria were prespecified: (1) animal models of myocardial I/R injury were induced by ligating of the left anterior descending coronary artery (LAD) or left coronary artery (LCA); (2) the treatment group was given with fasudil as monotherapy at any dose. Interventions for control group were isasteric and same solvent or no treatment; (3) the primary outcome measures were MI size and/or cardiac enzymes and/or cardiac troponin and/or the level of ST-segment depression and/or indicators which represent systolic and diastolic function of the heart in cardiac ultrasound. The second outcome measures were mechanisms of fasudil for myocardial I/R injury. There was no limitation on animal species. The exclusion criteria were prespecified as follows: (1) The experimental model was established in coronary heart disease model or myocardial infarction (MI) model or cell experiment model; (2) fasudil was not used as a monothrapy; (3) there was not a control group in the study; (4) the study was not published in peer-review journals. (5) the study was duplicate publication; (6) there was not predetermined outcome index in the study.

Two authors independently reviewed each included study and extracted following aspects of details: (1) name of first author, year of publication; (2) the detail information of animals for each study, including animal species, number, sex, and weight; (3) the use of anesthesia in the experiment and the methods to establish animal models; (4) the information of treatment and control group, including therapeutic drug dosage, method of administration, duration of treatment; (5) the outcome measures and samples for individual comparison were included. The data of highest dose and the result of the peak time point were selected for analysis when the treatment group included various doses of the target drug and the data were described at different times. The data of the preprocessing group were selected for analysis when the preprocessing group and the post-processing group were researched in a experiment. We made efforts to contact authors for further informations because some records' published data were only in graphical format. And the numerical values were measured from the graphs by digital ruler software when response was not received from authors.

### Risk of bias in individual studies

The methodological quality of each included study was evaluated by using Collaborative Approach to Meta-Analysis and Review of Animal Data from Experimental Studies (CAMARADES) 10-item checklist (Macleod et al., 2004) with minor modification as follows: A: peer-reviewed publication; B: control of temperature; C: random allocation to treatment or control; D: blinded induction of model (group randomly after the induction of ischemia); E: blinded assessment of outcome; F: use of anesthetic without significant intrinsic cardioprotective activity; G: appropriate animal model (aged, diabetic, or hypertensive); H: sample size calculation; I: compliance with animal welfare regulations [preoperative anesthesia, postoperative analgesia, nutrition, environment (temperature, humidity, circadian rhythm), and euthanasia]; and J: statement of potential conflict of interests. Every item was given one point. Two investigators independently evaluated the study quality and divergences were well settled through consulting with correspondence authors.

### Statistical analysis

The statistical analysis was conducted via Rev Man version 5.3, the bar graphs were drawn via Prism 6. A summary statistic was calculated for each comparison with 95% confidence intervals by using the random effects method. When the outcome measurements in all included studies in meta-analysis were based on the same scale, weighted mean difference (WMD) was calculated as a summary statistic. On the contrary, when the same outcome measurements were measured in a variety of ways across studies in meta-analysis, standardized mean difference (SMD) was used as a summary statistic. Heterogeneity between study results was investigated based on a standard chi-square test and *I*^2^ statistic. A fixed effects model (*I*^2^ < 50%) or a random effects model (*I*^2^ > 50%) was used depending on the value of *I*^2^. When probability value was less than 0.05 (type I error rate), the difference of the two groups was considered statistically significant.

## Results

### Study selection

**Figure 1:**
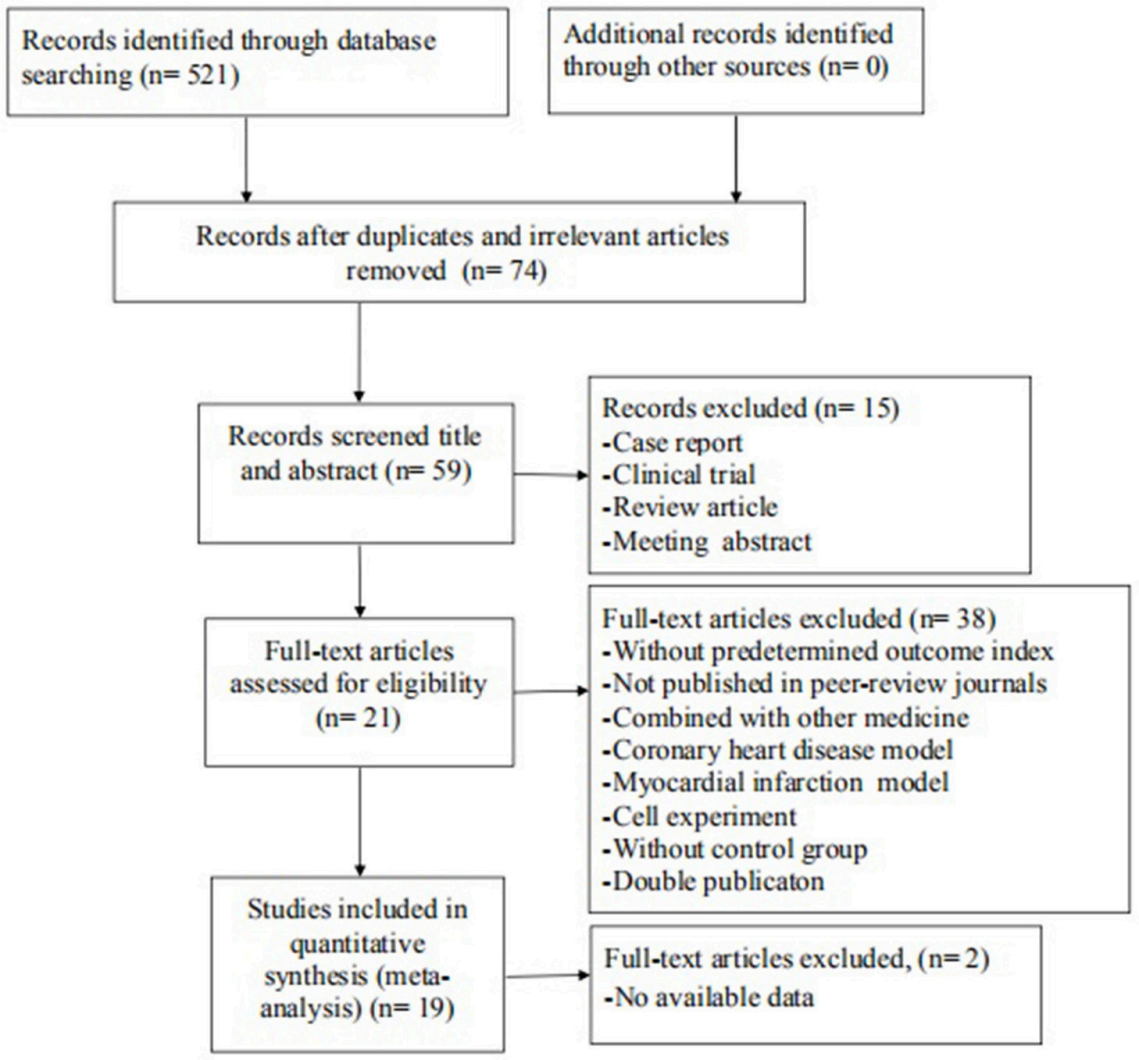
Summary of the process for identifying candidate studies.

The search strategy resulted in 521 unique citations, of which 447 were reduplicated and irrelevant articles.Of these, we included 59 articles after review of the title or abstract. After detailed examination, 38 articles were excluded if they met exclusion criteria. Then, we removed 2 articles in which data of result was not available. Ultimately, 19 articles (Hamid et al., 2007; Xie et al., 2007; Shibata et al., 2008; Zhang et al., 2009, 2018; Ichinomiya et al., 2012; Jiang et al., 2012, 2013; Li et al., 2012, 2014; Xi et al., 2012; Guan et al., 2013; Shang et al., 2013; Kitano et al., 2014; Lu et al., 2014; Wu et al., 2014; Deng et al., 2016; Ye et al., 2016; Min et al., 2017) were selected, Figure 1.

### Characteristics of included studies

Nine studies (Xie et al., 2007; Jiang et al., 2012; Xi et al., 2012; Guan et al., 2013; Shang et al., 2013; Lu et al., 2014; Deng et al., 2016; Ye et al., 2016; Min et al., 2017) were published in Chinese and 10 studies (Hamid et al., 2007; Shibata et al., 2008; Zhang et al., 2009, 2018; Ichinomiya et al., 2012; Li et al., 2012, 2014; Jiang et al., 2013; Kitano et al., 2014; Wu et al., 2014) in English between 2006 and 2018. Male/female Sprague Dawley (SD) rats were used in 12 studies (Hamid et al., 2007; Xie et al., 2007; Ichinomiya et al., 2012; Jiang et al., 2012, 2013; Li et al., 2012, 2014; Lu et al., 2014; Deng et al., 2016; Ye et al., 2016; Min et al., 2017; Zhang et al., 2018), male/female Wistar rats in 7 studies (Shibata et al., 2008; Zhang et al., 2009; Xi et al., 2012; Guan et al., 2013; Shang et al., 2013; Kitano et al., 2014; Wu et al., 2014), male C57BL/6J mice in 1 study (Kitano et al., 2014), male/female swine in 1 study (Shibata et al., 2008) and male New Zealand white rabbits in 1 study (Xi et al., 2012). To induce anesthesia, 4 studies (Guan et al., 2013; Li et al., 2014; Ye et al., 2016; Min et al., 2017) used chloral hydrate; 9 studies (Hamid et al., 2007; Xie et al., 2007; Zhang et al., 2009, 2018; Ichinomiya et al., 2012; Jiang et al., 2012; Li et al., 2012; Xi et al., 2012; Wu et al., 2014) used sodium pentobarbital; 1 study (Shibata et al., 2008) used both α-chloralose and fentanyl; and 1 study (Lu et al., 2014) used urethane, while anesthetic was not mentioned in the other 4 studies (Jiang et al., 2013; Shang et al., 2013; Kitano et al., 2014; Deng et al., 2016). All myocardial I/R models were established by ligation of the LAD. Among the dose use of fasudil, 1 study (Zhang et al., 2018) used 50 mg•kg^−1^; 8 studies (Xie et al., 2007; Zhang et al., 2009; Li et al., 2012, 2014; Shang et al., 2013; Kitano et al., 2014; Wu et al., 2014; Min et al., 2017) used 10 mg•kg^−1^; 1 study (Xi et al., 2012) used 6 mg•kg^−1^; 2 studies(Ichinomiya et al., 2012; Deng et al., 2016) used 0.5 mg•kg^−1^; 1 study (Shibata et al., 2008) used 13 μg•kg^−1^•min^−1^ for 30 min; 1 study (Jiang et al., 2012) used 500 μg•kg^−1^•min^−1^ for 5 min; 1 study (Jiang et al., 2013) used 500 μg/mL for 5 min; 1 study (Guan et al., 2013) used 10 μmol•L^−1^; 1 study (Lu et al., 2014) used 5 mg; 1 study (Ye et al., 2016) used 30 μmol•L^−1^; the remaining 1 study (Hamid et al., 2007) used 5 μmol•L^−1^ for 55 min. MI size was utilized as outcome measure in 14 studies (Hamid et al., 2007; Zhang et al., 2009, 2018; Ichinomiya et al., 2012; Jiang et al., 2012, 2013; Li et al., 2012, 2014; Guan et al., 2013; Shang et al., 2013; Kitano et al., 2014; Wu et al., 2014; Deng et al., 2016; Min et al., 2017), level of ST-segment elevation in 2 studies (Xi et al., 2012; Min et al., 2017), maximal rate of the increase/decrese of left ventricular pressure (±dp/dtmax) in 7 studies (Xie et al., 2007; Shibata et al., 2008; Jiang et al., 2012; Guan et al., 2013; Li et al., 2014; Wu et al., 2014; Ye et al., 2016), left ventricular developed pressure (LVDP) was reported in 4 studies (Guan et al., 2013; Li et al., 2014; Wu et al., 2014; Ye et al., 2016), left ventricular end-diastolic pressure (LVEDP) in 2 studies (Shibata et al., 2008; Ye et al., 2016), segment shortening in 1 study (Shibata et al., 2008), mean arterial pressure (MAP) in 2 studies (Shibata et al., 2008; Min et al., 2017), heart rate (HR) in 7 studies (Hamid et al., 2007; Shibata et al., 2008; Jiang et al., 2012; Guan et al., 2013; Li et al., 2014; Wu et al., 2014; Min et al., 2017). Myocardial cell apoptotic index was reported in 9 studies (Zhang et al., 2009, 2018; Jiang et al., 2012, 2013; Li et al., 2012, 2014; Kitano et al., 2014; Lu et al., 2014; Deng et al., 2016), Lactate dehydrogenase (LDH) in 6 studies (Xie et al., 2007; Jiang et al., 2012; Li et al., 2014; Wu et al., 2014; Ye et al., 2016; Min et al., 2017), creatine kinase (CK) in 3 studies (Xie et al., 2007; Jiang et al., 2012; Min et al., 2017), CK activity in 1 study (Zhang et al., 2018), creatine kinase-MB (CK-MB) in 3 studies (Xie et al., 2007; Xi et al., 2012; Wu et al., 2014), cardiac troponin I (cTnI) in 1 study (Xi et al., 2012). Bcl-2 and Bcl-2 associated X protein (Bax) was reported in 3 studies (Jiang et al., 2013; Li et al., 2014; Ye et al., 2016), caspase-3 in 4 studies (Li et al., 2012; Jiang et al., 2013; Ye et al., 2016; Zhang et al., 2018), concentration of Ca^2+^ in cardiac myocytes in 2 studies (Xie et al., 2007; Lu et al., 2014), superoxide dismutase (SOD) in 1 study (Guan et al., 2013), malondialdehyde (MDA) in 1 study (Guan et al., 2013), glyceraldehyde phosphate dehydrogenase (GAPDH) in 2 studies (Jiang et al., 2013; Kitano et al., 2014), tumor necrosis factor-α (TNF-α) in 2 studies (Xi et al., 2012; Kitano et al., 2014), interleukin-6 (IL-6) in 1 study (Kitano et al., 2014), interleukin-10 (IL-10) in 1 study (Kitano et al., 2014), endothelial nitric oxide synthase (eNOS) in 3 studies (Hamid et al., 2007; Li et al., 2014; Wu et al., 2014), NO in 3 studies (Xie et al., 2007; Xi et al., 2012; Shang et al., 2013), coronary blood flow (CBF) in 2 studies (Hamid et al., 2007; Shibata et al., 2008), myeloperoxidase (MPO) in 1 study (Xie et al., 2007), phosphothreonine kinase (p-Akt) in 4 studies (Li et al., 2012, 2014; Jiang et al., 2013; Wu et al., 2014). The overall characteristics of included studies are shown in Table 1.

**Table 1:** Characteristics of the 19 included studies.

**Study (years)**	**Species (Sex, *n* = experimental/control group)**	**Weight**	**Model (method)**	**Anesthetic**	**Treatment group (Method to astragal sides)**	**Control group**	**Outcome Index (time)**	**Intergroup differences**
Hamid et al., 2007	SD rats (male, 8/7)	250–350 g	Block LAD for 35 min then reflow for 120 min (Isolated rat hearts)	Sodium pentobarbital (50 mg/kg)	K-H solution with fasudil (5 μmol/l) was was pumped into the aortic root from 10 min before coronary occlusion until 10 min after reperfusion	K-H solution without fasudil injection was pumped into the aortic root during the process of experiment	1.Myocardial infarct size (IA/LVA) 2.HR 3.eNOS 4.CBF	1.*P* < 0.05 2.*P* < 0.05 3.*P* < 0.05 4.*P* < 0.05
Xie et al., 2007	SD rats (male/female, 6/7)	190–250 g	Block LAD for 45 min then reflow for 30 min (Isolated rat hearts)	Sodium pentobarbital (30 mg/kg)	K-H solution with fasudil injection (10 mg/kg for 30 min) was pumped into the aortic root 45 min after ischemia	K-H solution without fasudil injection (50 ml for 30 min) was pumped into the aortic root 45 min after ischemia	1.LDH 2.CK 3.CK-MB 4.NO 5.Ca^2+^ 6.±dp/dtm_ax_ 7.MPO	1.*P* < 0.05 2.*P* < 0.05 3.*P* < 0.05 4.*P* < 0.05 5.*P* < 0.05 6.*P* < 0.05 7.*P* < 0.05
Shibata et al., 2008	Swine (male/female, 10/11)	19–33 kg	Block LAD for 12 min then reflow for 90 min	α-chloralose (100 mg/kg), fentanyl (10 μg/kg)	By intravenous injection of fasudil (13 μg kg-1 min-1 for 30 min) until 15 min before establishing model	By intravenous injection of isasteric NS both before and after ischemia	1.CBF 2.HR 3.MAP 4. LVEDP 5.Segment shortening 6.±dp/dtmax 7. m-KATP	1.*P* < 0.05 2.*P* < 0.05 3.*P* < 0.05 4.*P* < 0.05 5.*P* < 0.05 6.*P* < 0.05 7.*P* < 0.05
Zhang et al., 2009	Wistar rats (female, 18/18)	250–300 g	Block LAD for 30 min then reflow for 180 min	Sodium pentobarbital (50 mg/kg)	By intravenous injection of fasudil (10 mg/kg) 60 min before establishing model	By intravenous injection of isasteric NS 60 min before establishing model	1.Myocardial infarct size (IA/LVA) 2.Apoptotic index 3.p-JNK2 4.m-AIF 5.n-AIF 6.MYPT-1 7. m-KATP	1.*P* < 0.05 2.*P* < 0.05 3.*P* < 0.05 4.*P* < 0.05 5.*P* < 0.05 6.*P* < 0.05 7.*P* < 0.05
Li et al., 2012	SD rats (male, 4/4)	250–280 g	Block LAD for 45 min then reflow for 24 h	Sodium pentobarbital (50 mg/kg)	By intravenous injection of fasudil (10 mg/kg) 15 min before establishing model	By intravenous injection of nothing before establishing model	1.Myocardial infarct size (IA/AR) 2.Apoptotic index 3.Caspase-3 4.p-Akt 5.p-JAK2 6. SERCA	1.*P* < 0.05 2.*P* < 0.05 3.*P* < 0.05 4.*P* < 0.05 5.*P* < 0.05 6. *P* < 0.05
Ichinomiya et al., 2012	SD rats (male, 10/10)	350–550 g	Block LAD for 30 min then reflow for 120 min	Sodium pentobarbital (50 mg/kg)	By intravenous injection of fasudil (0.5 mg/kg) 5 min before establishing model	By intravenous injection of isasteric NS 5 min before establishing model	1.Myocardial infarct size (IA/AR) 2.Blood glucose concentrations	1.*P* < 0.05 2.*P* < 0.05
Jiang et al., 2012	SD rats (male, 12/12)	240–260 g	Block LAD for 60 min then reflow for 120 min	Sodium pentobarbital (2 ml/kg, 3%)	By intravascular injection of fasudil (500 μg/(kg·min) for 5 min) into the coronary artery from aortic root before reperfusion	By intravascular injection of isasteric NS into the coronary artery from aortic root before reperfusion	1.Myocardial infarct size (IA/LVA) 2.Apoptosis indexs 3.CK 4.LDH 5.HR 6.±dp/dtm_ax_	1.*P* < 0.05 2.*P* < 0.01 3.*P* < 0.01 4.*P* < 0.05 5.*P* < 0.05 6.*P* < 0.05
Xi et al., 2012	New Zealand white rabbits (male, 12/12)	1.5–2.0 kg	Block LAD for 30 min then reflow for 90 min	Sodium pentobarbital (30 mg/kg)	By intravenous injection of fasudil (6 mg/kg) 30 min after establishing model	By intravenous injection of isasteric NS 30 min after establishing model	1.The level of ST-segment depression 2.CK-MB 3.cTnI 4.TNF-α 5.NO 6. m-KATP	1.*P* < 0.05 2.*P* < 0.05 3.*P* < 0.05 4.*P* < 0.05 5.*P* < 0.05 6.*P* < 0.05
Jiang et al., 2013	SD rats (male, 18/18)	240–260 g	Block LAD for 60 min then reflow for 120 min (Isolated rat hearts)	Not mentioned	Fasudil (500 μg/mL/min for 5 min) was pumped into the aortic root, followed by reperfusion for 115 min	Blood was pumped into the aortic root during the process of experiment	1.Myocardial infarct size (IA/LVA) 2.Apoptotic index 3.Bcl-2 4.Bax 5.Caspase-3 6.P-Akt 7.GAPDH 8.ROCK	1.*P* < 0.05 2.*P* < 0.05 3.*P* < 0.05 4.*P* < 0.05 5.*P* < 0.05 6.*P* < 0.05 7.*P* < 0.05 8.*P* < 0.05
Shang et al., 2013	Wistar rats (male, 10/10)	250–300 g	Block LAD for 30 min then reflow for 24 h	Not mentioned	By intraperitoneal injection of fasudil (10 mg/kg) 60 min before establishing model	By intraperitoneal injection of isasteric NS 60 min before establishing model	1.Myocardial infarct size (IA/LVA) 2.NO 3.VWF	1.*P* < 0.05 2.*P* < 0.05 3.*P* < 0.05
Guan et al., 2013	Wistar rats (male, 10/10)	250–300 g	Block LAD for 30 min then reflow for 120 min (Isolated rat heart)	Chloral hydrate (10%)	K-H solution with fasudil (10 μmol/L) was poured into coronary 15 min before establishing model	K-H solution without fasudil was poured into coronary during the process of experiment	1.Myocardial infarct size (IA/LVA) 2.MDA 3.SOD 4.HR 5.LVDP 6.±dp/dtm_ax_	1.*P* < 0.05 2.*P* < 0.01 3.*P* < 0.01 4.*P* < 0.05 5.*P* < 0.05 6.*P* < 0.05
Wu et al., 2014	Wistar rats (male, 10/10)	190–210 g	Block LAD for 30 min then reflow for 120 min (Isolated rat hearts)	Sodium pentobarbital (100 mg/kg)	K-H solution with fasudil (10 mg/kg) was poured into coronary 15 min before establishing model	K-H solution without fasudil was poured into coronary during the process of experiment	1.Myocardial infarct size (IA/LVA) 2.CK-MB 3.LDH 4.p-Akt 5.eNOS 6.HR 7.LVDP 8.±dp/dtm_ax_	1.*P* < 0.05 2.*P* < 0.05 3.*P* < 0.05 4.*P* < 0.05 5.*P* < 0.05 6.*P* < 0.05 7.P > 0.05 8.P > 0.05
Kitano et al., 2014	C57BL/6J mice (male, 15/15)	Not mentioned	Block LAD for 30 min then reflow for 24 h	Not mentioned	By intraperitoneal injection of fasudil (10 mg/kg) 60 min before establishing model	By intraperitoneal injection of isasteric NS 60 min before establishing model	1.Myocardial infarct size (IA/AR) 2.Apoptotic index 3.IL-6 4.TNF-α 5.IL-10 6.MYPT-1 7.GAPDH	1.*P* < 0.05 2.*P* < 0.05 3.*P* < 0.05 4.*P* < 0.05 5.*P* < 0.05 6.*P* < 0.05 7.*P* < 0.05
Li et al., 2014	SD rats (male, 10/10)	250–300 g	Block LAD for 30 min then reflow for 120 min (Isolated rat hearts)	Chloral hydrate (10%, 4 ml/kg)	By intraperitoneal injection of fasudil (10 mg/kg) 60 min before establishing model	By intraperitoneal injection of isasteric NS 60 min before establishing model	1.Myocardial infarct size (IA/LVA) 2.Apoptosis indexs 3.LDH 4.Bcl-2 5.Bax 6.eNOS 7.P-Akt 8.HR 9.LVDP 10.±dp/dtm_ax_	1.*P* < 0.05 2.*P* < 0.05 3.*P* < 0.05 4.*P* < 0.05 5.*P* < 0.05 6.*P* < 0.05 7.*P* < 0.05 8.*P* < 0.05 9.*P* < 0.05 10.*P* < 0.05
Lu et al., 2014	SD rats (male, 15/15)	250–350 g	Block LAD for 30 min then reflow for 120 min	Not mentioned	By intravenous injection of fasudil (5 mg) 30 min before establishing model	By intravenous injection of NS (2 ml/100 g) 30 min before establishing model	1.Apoptosis indexs 2.Ca^2+^ 3.MYPT1	1.*P* < 0.01 2.*P* < 0.01 3.*P* < 0.01
Ye et al., 2016	SD rats (male, 8/8)	220–250 g	Block LAD for 30 min then reflow for 120 min (Isolated rat hearts)	Chloral hydrate (4%)	K-H solution with fasudil injection (30 μmol/L) was pumped into the aortic root 10 min earlier before establishing model until 10 min after reperfusion	K-H solution without fasudil injection was pumped into the aortic root during the process of experiment	1.LDH 2.Bax 3.Bcl-2 4.Caspase-3 5.LVEDP 6.LVDP 7.±dp/dtm_ax_	1.*P* < 0.05 2.*P* < 0.05 3.*P* < 0.05 4.*P* < 0.05 5.*P* < 0.05 6.*P* < 0.05 7.*P* < 0.05
Deng et al., 2016	SD rats (male, 5/5)	225–271 g	Block LAD for 30 min then reflow for 120 min	Not mentioned	By intravenous injection of fasudil (0.5 mg/kg) before reperfusion	By intravenous injection of nothing before reperfusion	1.Myocardial infarct size (IA/AR) 2.Apoptosis indexs	1.*P* < 0.01 2.*P* < 0.01
Min et al., 2017	SD rats (male, 6/6)	250–350 g	Block LAD for 45 min than reflow for 180 min	Chloral hydrate (10 mL/kg, 4%)	By intravenous injection of fasudil (10 mg/kg) 5 min before reperfusion	By intravenous injection of nothing before reperfusion	1.Myocardial infarct size (IA/AR) 2.ST-segmen televation 3.MAP 4.HR 5.CK 6.LDH 7.p-MLC	1.*P* < 0.05 2.*P* < 0.01 3.*P* < 0.01 4.*P* < 0.05 5.*P* < 0.05 6.*P* < 0.05 7.*P* < 0.05
Zhang et al., 2018	SD rats (male, 8/8)	210–250 g	Block LAD for 60 min then reflow for 180 min	Sodium pentobarbital (60 mg/kg)	By intraperitoneal injection of fasudil (50 mg/kg) before reperfusion	By intraperitoneal injection of nothing before reperfusion	1.Myocardial infarct size (general views of the naked eye) 2.Apoptosis indexs 3.CK activity 4. Caspase-3 5. Real-time PCR 6.Hydrogen peroxide	1.*P* < 0.05 2.*P* < 0.05 3.*P* < 0.05 4.*P* < 0.05 5.*P* < 0.05 6.*P* < 0.05

*AST, aspartate transaminase; Bax, Bcl-2 associated X protein; CBF, Coronary blood flow; CK, creatine kinase; CK-MB, creatine kinase-MB; CPK, creatine phosphokinase; MPO, myeloperoxidase; cTnI, cardiac troponin I; cTnT, cardiac troponin T; GSH, glutathione synthetase; HR, heart rate; HSP, heat shock protein; LAD, the left anterior descending coronary artery; LDH, lactate dehydrogenase; LVSP, left ventricular systolic pressure; LVDP, left ventricular developed pressure; LVEDP, left ventricular end-diastolic pressure; IA/AR, infarct area/area at risk; IA/LVA, infarct area/left ventricular area; K-H solution, Krebs-Henseleit solution; MAP, mean arterial pressure; MDA, malondialdehyde; MYPT-1, myosin phosphatase target subunit 1; NS, normal saline; p-Akt, phosphorylated Akt; ROCK, Rho associated coiled-coil forming protein kinase; SD rats, Sprague-Dawley; SOD, superoxide dismutase; TNF-α, tumor necrosis factor-α; VF, ventricular fibrillation; VT, ventricular tachycardia; VWF, von Willebrand factor; SERCA, sarco endoplasmic reticulum calcium adenosine triphosphatase; m-KATP, mitochondrial KATP*.

### Study quality

The quality scores of studies ranged from 3 to 6 in a total of 10 points. All the included studies were publications in a peer reviewed journals and all animals were allocated randomly to treatment or control. However, none of the studies described a sample size calculation, blinded induction of model or blinded assessment of outcome. Sixteen studies (Hamid et al., 2007; Xie et al., 2007; Shibata et al., 2008; Zhang et al., 2009, 2018; Ichinomiya et al., 2012; Jiang et al., 2012; Li et al., 2012, 2014; Guan et al., 2013; Shang et al., 2013; Kitano et al., 2014; Wu et al., 2014; Deng et al., 2016; Ye et al., 2016; Min et al., 2017) reported control of temperature. Fifteen studies declared use of anesthetic without significant intrinsic vascular protection activity; 12 studies (Hamid et al., 2007; Zhang et al., 2009, 2018; Ichinomiya et al., 2012; Jiang et al., 2012, 2013; Li et al., 2012, 2014; Guan et al., 2013; Kitano et al., 2014; Wu et al., 2014; Deng et al., 2016) mentioned no potential conflict of interests, and 5 studies (Zhang et al., 2009, 2018; Jiang et al., 2012, 2013; Lu et al., 2014) described compliance with animal welfare regulations. Only 2 studies (Ichinomiya et al., 2012; Wu et al., 2014) adpoted rats with hyperglycemia or hypercholesterolemia, and others adpoted healthy rats. The methodological quality is concluded in Table 2.

**Table 2:** Risk of bias of the included studies.

**Study**	**A**	**B**	**C**	**D**	**E**	**F**	**G**	**H**	**I**	**J**	**Total**
Hamid et al., 2007	√	√	√			√				√	5
Xie et al., 2007	√	√	√			√					4
Shibata et al., 2008	√	√	√			√					4
Zhang et al., 2009	√	√	√			√			√	√	6
Li et al., 2012	√	√	√			√				√	5
Ichinomiya et al., 2012	√	√	√			√	√			√	6
Jiang et al., 2012	√	√	√			√			√	√	6
Xi et al., 2012	√		√			√					3
Jiang et al., 2013	√		√						√	√	4
Shang et al., 2013	√	√	√								3
Guan et al., 2013	√	√	√			√				√	5
Wu et al., 2014	√	√	√			√	√			√	6
Kitano et al., 2014	√	√	√			√				√	5
Li et al., 2014	√	√	√			√				√	5
Lu et al., 2014	√		√						√		3
Ye et al., 2016	√	√	√			√					4
Deng et al., 2016	√	√	√							√	4
Min et al., 2017	√	√	√			√					4
Zhang et al., 2018	√	√	√			√			√	√	6

*Studies fulfilling the criteria of: A, peer reviewed publication; B, control of temperature; C, random allocation to treatment or control; D, blinded induction of model (group randomly after the induction of ischemia); E, blinded assessment of outcome; F, use of anesthetic without significant intrinsic vascular protection activity; G, appropriate animal model (aged, diabetic, or hypertensive); H, sample size calculation; I, compliance with animal welfare regulations [preoperative anesthesia, postoperative analgesia, nutrition, environment (temperature, humidity, circadian rhythm), and euthanasia]; J, statement of potential conflict of interests*.

### Effectiveness

#### MI size

**Figure 2:**
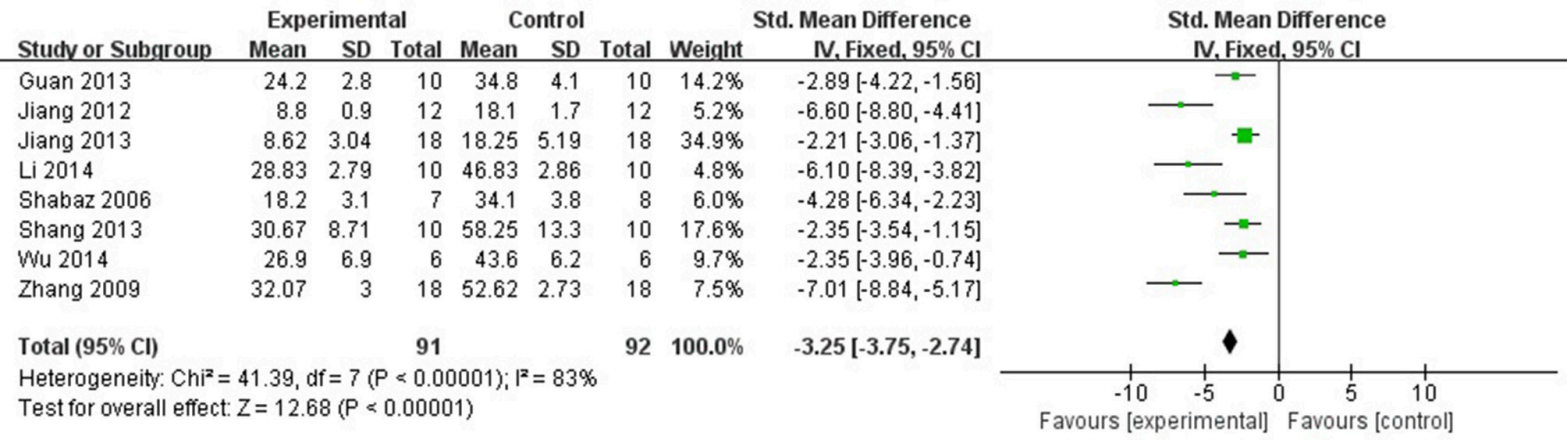
The forest plot: effects of fasudil for decreasing infarct area/left ventricular area compared with control group.

Eight studies (Hamid et al., 2007; Zhang et al., 2009; Jiang et al., 2012, 2013; Guan et al., 2013; Shang et al., 2013; Li et al., 2014; Wu et al., 2014) reported the MI size (infarct area/left ventricular area) as the outcome measure included in the analysis. We pooled the whole data to process and found a significant difference in favor of fasudil for decreasing infarct area/left ventricular area compared with control group (*n* = 183, SMD −3.25, 95% CI: −3.75 ~ −2.74, *P* < 0.00001;χ^2^ = 41.39, *P* < 0.00001, *I*^2^ = 83%), Figure 2. Sensitivity analyses showed that the results did not substantially alter after removing any 1 study.

**Figure 3:**
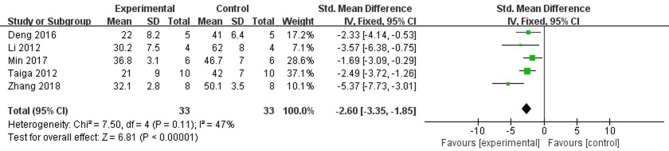
The forest plot: effects of fasudil for decreasing the infarct area/area at risk compared with control group.

Six studies (Ichinomiya et al., 2012; Li et al., 2012; Kitano et al., 2014; Deng et al., 2016; Min et al., 2017; Zhang et al., 2018) reported the MI size (infarct area/area at risk) as the outcome measure. After sensitivity analyses, we removed 1 study (Kitano et al., 2014) that MI experimental model was established in C57BL/6J mice, unlike other studies using rats. Meta-analysis of 5 studies (Ichinomiya et al., 2012; Li et al., 2012; Deng et al., 2016; Min et al., 2017; Zhang et al., 2018) showed a significant difference in favor of fasudil for decreasing the infarct area/area at risk compared with control group (*n* = 66, SMD −2.60, 95% CI: −3.35 ~ −1.85, *P* < 0.00001;χ^2^ = 7.50, *P* < 0.00001, *I*^2^ = 47%), Figure 3. Significant decreases of the infarct area/area at risk in fasudil group was also reported in the remaining 1 study (Kitano et al., 2014) (*P* < 0.05).

#### Systolic and diastolic function of the heart in cardiac ultrasound and the level of ST-segment depression in electrocardiogram

For systolic function, fasudil can increase +dp/dtmax (Xie et al., 2007; Shibata et al., 2008; Jiang et al., 2012; Guan et al., 2013; Li et al., 2014; Ye et al., 2016), and segment shortening (Shibata et al., 2008) compared with control (*P* < 0.05). For diastolic function, fasudil can increase -dp/dtmax (Xie et al., 2007; Shibata et al., 2008; Jiang et al., 2012; Guan et al., 2013; Li et al., 2014; Ye et al., 2016), LVDP (Guan et al., 2013; Li et al., 2014; Ye et al., 2016), and decrease LVEDP (Shibata et al., 2008; Ye et al., 2016) compared with control (*P* < 0.05). In addition, two studies (Xi et al., 2012; Min et al., 2017) reported that fasudil can increase degree of decline in the ST-segment in electrocardiogram compared with control (*P* < 0.05).

#### Cardiac enzymes and/or cardiac troponin

**Figure 4:**
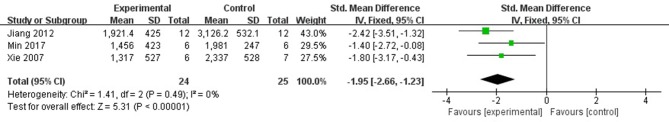
The forest plot: effects of fasudil for reducing creatine kinase compared with control group.

Six studies (Xie et al., 2007; Jiang et al., 2012; Li et al., 2014; Wu et al., 2014; Ye et al., 2016; Min et al., 2017) used LDH as a outcome measure. They failed to pool analysis because 3 studies (Xie et al., 2007; Jiang et al., 2012; Min et al., 2017) was set up in isolated heart model while the other 3 studies was set up *in vivo* (Li et al., 2014; Wu et al., 2014; Ye et al., 2016). But they all reported the significant effects of fasudil for reducing LDH compared with control group (*P* < 0.05 or *P* < 0.01). Meta-analysis of 3 studies (Xie et al., 2007; Jiang et al., 2012; Min et al., 2017) showed significant effect of fasudil for reducing CK compared with control group (*n* = 20, SMD −4.43, 95% CI (−5.68 to −3.17), *P* < 0.00001; heterogeneity: χ^2^ = 0.79, *df* = 1 (*P* = 0.49); *I*^2^ = 0%), Figure 4. Fasudil in 3 studies (Xie et al., 2007; Xi et al., 2012; Wu et al., 2014) showed the significant effects for reducing CK-MB compared with control (*P* < 0.05 or *P* < 0.01) according to CK-MB as the outcome measure. Only 1 study (Xi et al., 2012) showed that fasudil had a significant effect to reduce cTnT compared with control group (*P* < 0.05).

### Subgroup analysis

**Figure 5:**
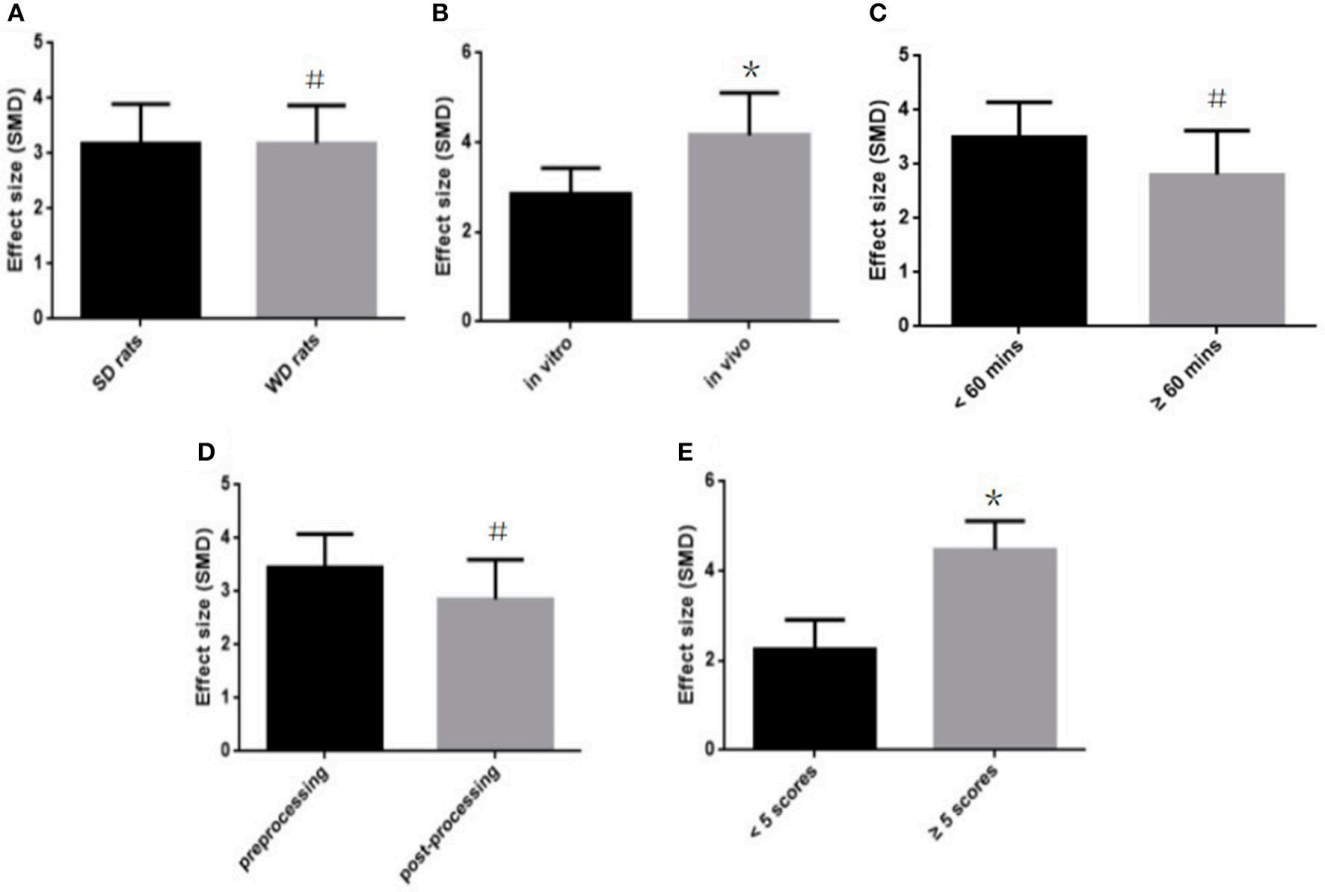
Subgroup analyses of the myocardial infarct size (infarct area/left ventricular area). **(A)** The animal species on the effect size of the outcome measure; **(B)** the animal model on the effect size of the outcome measure; **(C)** the infarct time on the effect size of the outcome measure; **(D)** the pretreatment and postconditioning on the effect size of the outcome measure; **(E)** the quality of studies on the effect size of the outcome measure. the magnitude of absolute value SMD reflected the effect size. *P < 0.05 vs. control groups; ^#^*P* > 0.05 vs. control groups.

To explore potential confounding factors that affected the outcome measures, we analyzed the MI size (infarct area/left ventricular area) in different subgroups stratified on the basis of the following variables: different animal (SD rats or Wistar rats), experimental model (*in vitro* or *in vivo*), infarct time, stage of fasudil administration (pre-processing and post-processing), and the quality of study. No difference was seen between the infarct sizes of the SD and Wistar rats (SMD = −3.27 vs. SMD = −3.22, *P* > 0.05, Figure 5A). Different experimental models varied considerably in the overall outcomes, and the model *in vivo* showed a better outcome compared with the model *in vitro* (SMD = −4.12 vs. SMD = −2.87, *P* < 0.05, Figure 5B). The infarct time (< 60 min or ≧60 min) had no significant effect on the size (SMD = −3.56 vs. SMD = −2.79, *P* > 0.05, Figure 5C). In addition, fasudil pre-treatment (prior to establishing the MI model) also did not affect infarct size compared to when fasudil was given after establishing the model (SMD = −3.48 vs. SMD = −2.98, *P* > 0.05, Figure 5D). Finally, the lower quality studies did not exhibit larger effect size than those with higher quality (SMD = −2.26 vs. SMD = −4.34, *P* < 0.05, Figure 5E).

## Discussion

### Summary of evidence

This is the first systematic review of the preclinical studies available in English and Chinese language that determines the efficacy and possible mechanisms of fasudil for myocardial I/R injury. The evidence of 19 eligible studies involving 400 animals shows a cardio-protective role of fasudil in animal models of myocardial I/R injury, through different mechanisms such as improvement in coronary vasodilation, inhibition of apoptosis, inflammation and oxidative stress, and reduction of endoplasmic reticulum stress.

### Limitations

We only reviewed English and Chinese language articles from 8 frequently-used databases, and the absence of studies in other languages or from other databases may have generated selective bias to a certain degree. Secondly, not all studies included the information regarding calculation of sample size, blindness of model establishment and outcome measurement. In addition, except for 2 studies (Ichinomiya et al., 2012; Wu et al., 2014) that used rats with hyperglycemia or hypercholesterolemia, none of other studies used animals with relevant comorbidities or risk factors (i.e., age, diabetes, atherosclerosis, hypercholesterolemia, hyperglycemia or hypertension) which are often observed in clinical AMI patients (Blankstein et al., 2012). Finally, only 1 or 2 studies supported a specific mechanism of fasudil action which need further validation.

### Implications

Isolated heart models are used to evaluate the cardio-protective efficacy of various drugs and analyze different biological parameters, thereby avoiding the influence of systematic factors *in vivo*, and imparting a high level of reproducibility in the experiments. In addition, isolated heart models have the additional advantage of regulating both pre-load and after-load on the heart, and allow a low-flow ischemic state. Finally, it is possible to obtain the electrical signal directly from the heart surface in the absence of other tissues that can affect signal transduction, in order to locate the ectopic foci of electrical activity and elucidate the basis of inducing arrhythmias (Chou et al., 2005; Wang et al., 2013). However, in our subgroup analysis, the *in vivo* heart model showed greater effect on the infarct size compared to the isolated heart model (SMD = −4.12 vs. SMD = −2.87, *P* < 0.05). We also stratified analysis of the MI size (infarct area/area at risk) and the myocardial cell apoptotic index based on *in vitro* and *in vivo* models. The *in vivo* heart model had greater effect on the myocardial cell apoptotic index than the isolated heart model (SMD = −3.57 vs. SMD = −1.94, *P* < 0.05). Studies using infarct area/area at risk as outcome measure failed to pool subgroup analysis because they all were performed *in vivo* model. There are several reasons that can explain the limitations of the isolated heart model. The foremost reason is that this model cannot completely simulate the complexity of the cardiac environment. Secondly, the model has to be prepared rapidly and gently to avoid cardiac injury and ATP depletion because of ischemia (Döring, 1990), along with maintaining the optimal experimental conditions (e.g., nutrient delivery, oxygen, perfusion pressure and temperature) to ensure reproducibility (Skrzypiec et al., 2007; Stensløkken et al., 2009), which makes the procedure technically demanding. Finally, use of protein-free solutions in long-term experiments causes tissue edema, resulting in the decline of cardiac contractile and chronotropic functions (Walters et al., 1992; Mouren et al., 2010).

Although the pre-processing group showed greater effect than the post-processing group, there was no significant difference (SMD = −3.48 vs. SMD = −2.98, *P* > 0.05). Considering that it is a comparison between different experiments, we have perused all included studies and compiled studies which were designed to investigate the differences of preventive and therapeutic effects of fasudil for myocardial I/R injury in the same experiments. Of which, 2 studies (Guan et al., 2013; Li et al., 2014) showed better effect of the pre-processing group (*P* < 0.05), 1 study (Wu et al., 2014) showed no difference (*P* > 0.05), and 1 study (Shibata et al., 2008) showed that administration of fasudil before ischemia or just after reperfusion, but not 30 min after reperfusion, protected the stunned myocardium. With a half-life of less than 15 min, fasudil is quickly cleared from the bloodstream, whereas its hydroxylated metabolite hydroxyfasudil, which preferentially inhibits Rho-kinase, remains in the blood for as long as 8 h after infusion (Nakashima et al., 1992). Therefore, we hypothesized that the cardio-protective effect of fasudil administered before ischemia is exerted through a mechanism other than maintenance of hydroxyfasudil during reperfusion. In addition, most injuries responsible for myocardial stunning and infarction develop during the early phase of reperfusion (Bolli and Marbán, 1999). Hamid et al. (2007) reported that Rho-kinase activity increased 10 min after reperfusion but not during ischemia, and that administration of another Rho-kinase inhibitor Y27632 during early reperfusion significantly reduced the MI size. In the study of Wu et al. (2014), fasudil was administered just after reperfusion, which is consistent with the above theory. Therefore, administration of fasudil before ischemia and during early reperfusion can protect against myocardial I/R injury.

Lower-quality trials often pool statistically significant 30–50% exaggeration of treatment efficacy when compared to higher-quality trials (Moher et al., 1998), although no overestimation of effect size was observed in the lower-quality studies in our subgroup analysis (SMD = −2.26 vs. SMD = −4.34, *P* < 0.05). In our study, the quality of included studies was considered moderate, and the scores ranged from 3 to 6 out of a total of 10 points. Several domains had flaws, especially in blinding, appropriate animal model and sample size calculation, which are the core standards of study design (Moher et al., 2015). Poor experimental designing is a major roadblock in translating the findings in animal models into promising pre-clinical and clinical trials on humans (Hackam and Redelmeier, 2006). Therefore, we recommend that these studies should design their experiments and report the results according to the ARRIVE guidelines (Kilkenny et al., 2012), with major focus on sample size calculation, blinding, and using the appropriate animal model. In addition, the different points related to animal treatment, such as pre-operative anesthesia, post-operative analgesia, nutrition, environment (temperature, humidity, circadian rhythm) and euthanasia should be documented in detail, since lack of humane treatment of the animals may affect the accuracy of the results (Kilkenny et al., 2010).

Animal experiments can contribute to our understanding of the mechanisms of various diseases (Hackam and Redelmeier, 2006). Based on our findings, the possible mechanisms of fasudil mediated cardio-protection are as follows: (1) improving coronary vasodilation by activating the PI3K/Akt signaling pathway (Shibata et al., 2008; Ichinomiya et al., 2012; Li et al., 2012, 2014; Jiang et al., 2013; Shang et al., 2013; Wu et al., 2014; Ye et al., 2016; Min et al., 2017) to up-regulate eNOS expression (Xie et al., 2007; Shibata et al., 2008; Ichinomiya et al., 2012; Xi et al., 2012; Shang et al., 2013; Li et al., 2014; Wu et al., 2014) and down-regulate MPO (Xie et al., 2007), thereby enhance NO production (Xie et al., 2007; Xi et al., 2012; Shang et al., 2013; Min et al., 2017); (2) inhibiting the phosphorylation of phosphorylated myosin light chain (p-MLC) (Min et al., 2017) and myosin phosphatase target subunit 1 (MYPT-1) (Zhang et al., 2009; Kitano et al., 2014; Lu et al., 2014), which lowers calcium ion concentration in the cytoplasm of vascular smooth muscle cells (Chen et al., 2013) to alleviate coronary artery spasms; (3) inhibiting apoptosis by up-regulating Bcl-2 (Li et al., 2014; Ye et al., 2016) and downregulating Bax (Li et al., 2014; Ye et al., 2016) and caspase-3 (Ye et al., 2016), which decreases Ca^2+^ release (Lu et al., 2014) and suppresses c-Jun NH2-terminal kinase-mediated apoptosis-inducing factor (AIF) translocation (Zhang et al., 2009); (4) inhibiting the inflammatory response by blocking IL-6 (Kitano et al., 2014), IL-10 (Kitano et al., 2014) and TNF-α (Xi et al., 2012; Kitano et al., 2014) production; (5) reversing the oxidative stress by enhancing SOD activity via attenuation of chondriokinesis, which lowers MDA release and NADPH expression (Zhang et al., 2018); (6) reducing endoplasmic reticulum stress and modulating sarco endoplasmic reticulum calcium adenosine triphosphatase (SERCA) activity via activation of the JAK2/STAT3 signaling pathway (Li et al., 2012); (7) competing with ATP for ATP binding sites in the Rho kinase catalytic domain (Jiang et al., 2012); (8) enhancing the activation of the mitochondrial KATP (m-KATP) channel (Ichinomiya et al., 2012; Xi et al., 2012; Wu et al., 2014); (9) improving autophagy by up-regulating autophagy-related gene 5 (Atg5) and Beclin1 mRNAs (Ye et al., 2016), Figure 6.

**Figure 6:**
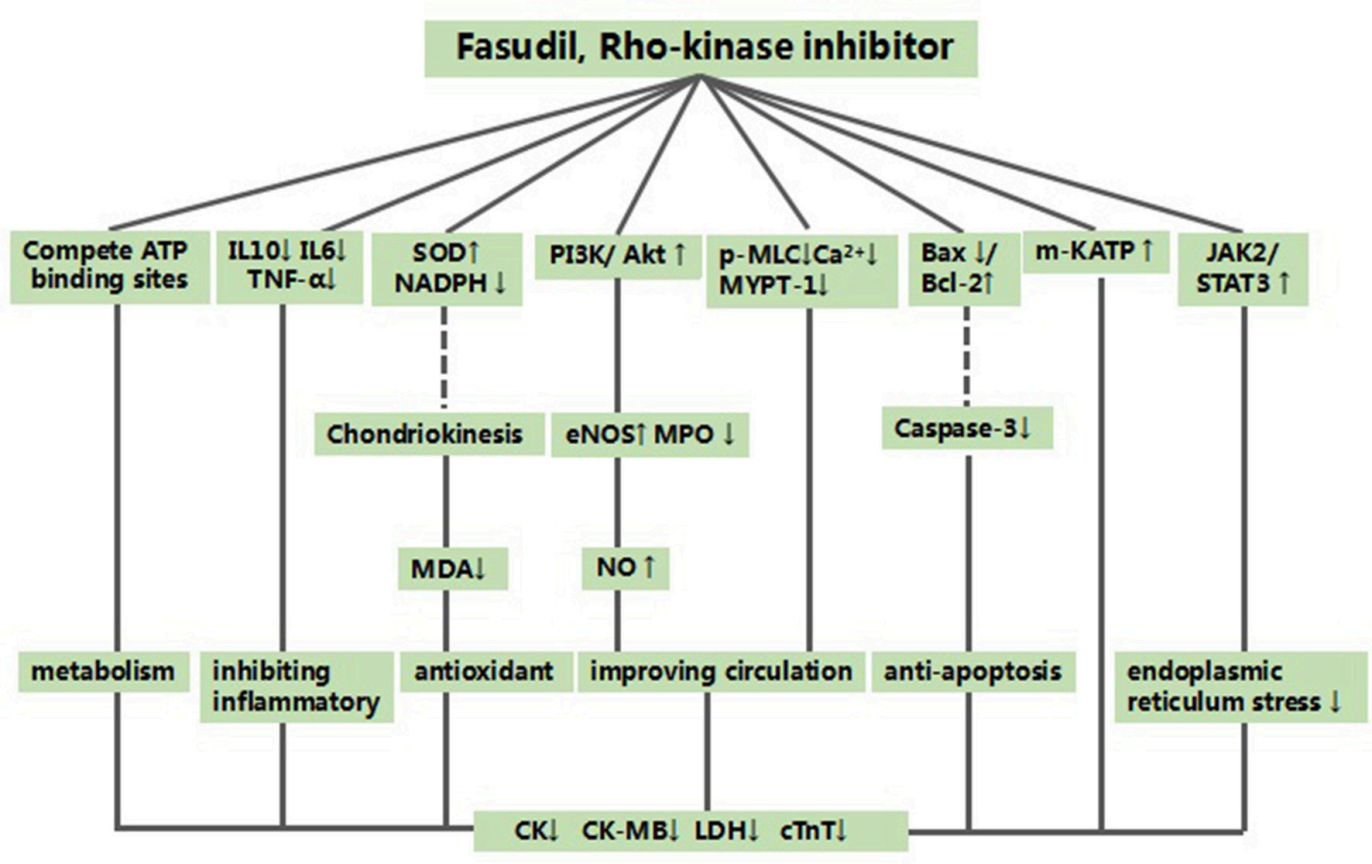
A schematic representation of cardioprotective mechanisms of fasudil for myocardial ischemia/reperfusion injury. Solid lines indicate established effects, whereas dashed lines represent putative mechanisms. SD rats, Sprague-Dawley rats; WD rats, Wistar rats.

Based on the above, we propose fasudil as a potential therapeutic option for patients with myocardial I/R injury. Unlike most drugs that are still in the pre-clinical stage, fasudil has been used clinically on cerebral vasospasm for many years with less adverse reactions (Satoh et al., 2011; Shi and Wei, 2013). Secondly, fasudil and its metabolites have favorable pharmacokinetic characteristics, such as rapid metabolism, wide distribution, water solubility, and effective oral administration (Chen et al., 2013). Finally, inhibitors of the Rho-ROCK signaling pathway are considered highly “drugable” and important targets for the treatment of cardiovascular diseases (Nunes et al., 2010; Satoh et al., 2011). In view of the disparities between the animal studies and the clinical trials, rigorous high-quality RCTs are needed to accurately assess the safety and efficacy of fasudil for patients with myocardial I/R injury.

## Conclusion

Administration of fasudil before ischemia and during early reperfusion is cardio-protective in animal models of myocardial I/R injury. The possible mechanisms of fasudil mediated cardio-protection are improved coronary vasodilation, inhibition of oxidative stress, inflammatory response and apoptosis, and increased angiogenesis. Therefore, fasudil is a potential cardio-protective candidate for further clinical trials on myocardial I/R injury.

## Author contributions

YH, JW, SZ, and XL designed the study. JW and TS collected the data. YH and JW performed all analyses. YH, JW, and XL wrote the manuscript. All authors contributed to the writing of this manuscript.

### Conflict of interest statement

The authors declare that the research was conducted in the absence of any commercial or financial relationships that could be construed as a potential conflict of interest. The reviewer CF and handling editor declared their shared affiliation at the time of the review.
